# Supplementary material for: Venomix: a simple bioinformatic pipeline for identifying and characterizing toxin gene candidates from transcriptomic data
Source: PeerJ. 2018 Jul 31;6:e5361. doi: 10.7717/peerj.5361 (PMC6074769; doi:10.7717/peerj.5361)
Supplement: Supplemental Information 3 [file peerj-06-5361-s003.gz › FinalOutput_E-6/Zinc_metalloproteinase@disintegrin_4/finaltree.pdf]

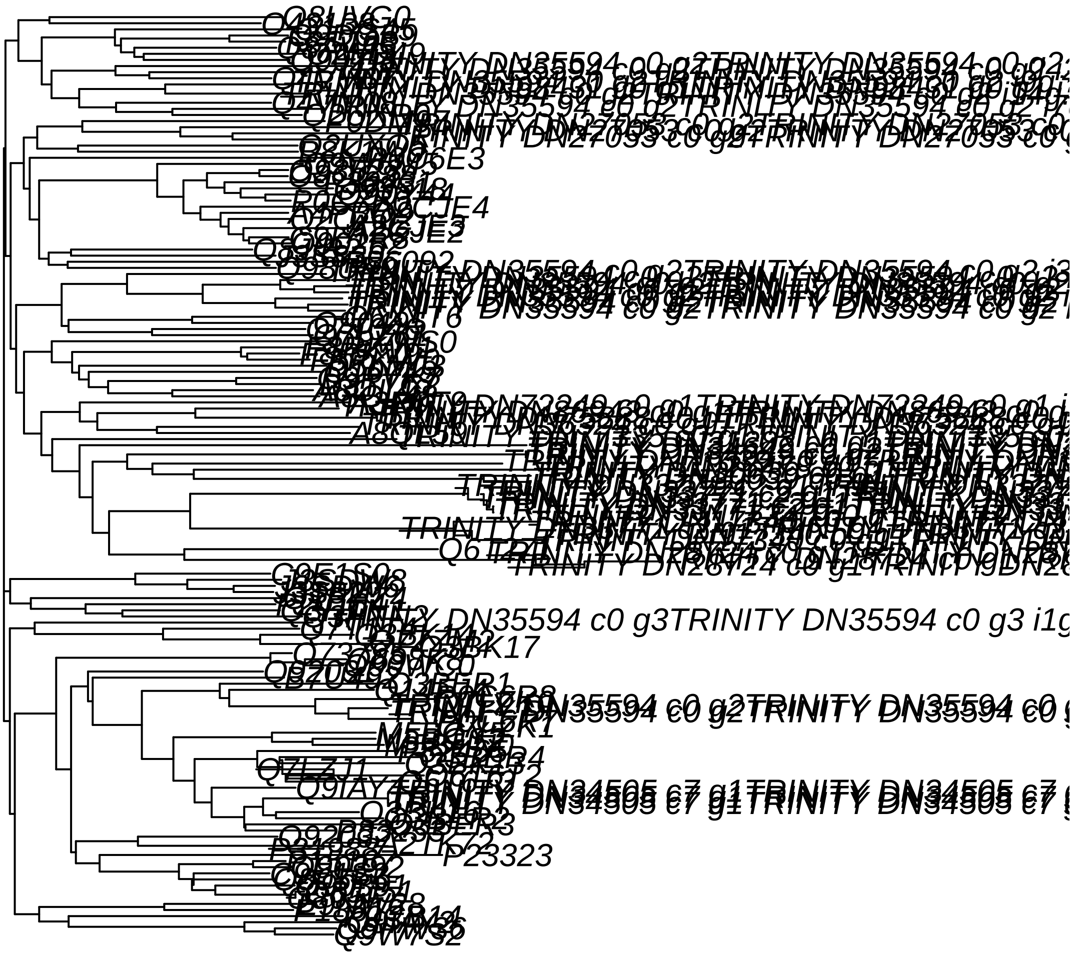

Q8UVG0
TRINITY DN35594 c0 g3TRINITY DN35594 c0 g3 i1g
P23323
Q9WYS2
